# Immune reconstitution inflammatory syndrome presenting as chylothorax in a patient with HIV and *Mycobacterium tuberculosis *coinfection: a case report

**DOI:** 10.1186/1471-2334-10-321

**Published:** 2010-11-08

**Authors:** Jiun-Nong Lin, Chung-Hsu Lai, Yen-Hsu Chen, Lin-Li Chang, Susan Shin-Jung Lee, Hsi-Hsun Lin

**Affiliations:** 1 Division of Infectious Diseases, Department of Internal Medicine, E-Da Hospital/I-Shou University, Kaohsiung, Taiwan; 2 Department of Critical Care Medicine, E-Da Hospital/I-Shou University, Kaohsiung, Taiwan; 3 Graduate Institute of Medicine, College of Medicine, Kaohsiung Medical University, Kaohsiung, Taiwan; 4 Tropical Medicine Research Institute, College of Medicine, Kaohsiung Medical University, Kaohsiung, Taiwan; 5 Division of Infectious Diseases, Department of Internal Medicine, Kaohsiung Medical University Hospital, Kaohsiung, Taiwan; 6 Division of Infectious Diseases, Department of Internal Medicine, Kaohsiung Veterans General Hospital, Kaohsiung, Taiwan; 7 Institute of Clinical Medicine, National Yang-Ming University, Taipei, Taiwan

## Abstract

**Background:**

Patients with human immunodeficiency virus (HIV) infection are at risk for *Mycobacterium tuberculosis *(TB) coinfection. The advent of antiretroviral therapy restores immunity in HIV-infected patients, but predisposes patients to immune reconstitution inflammatory syndrome (IRIS).

**Case Presentation:**

A 25-year-old HIV-infected male presented with fever, productive cough, and body weight loss for 2 months. His CD4 cell count was 11 cells/μl and HIV-1 viral load was 315,939 copies/ml. Antituberculosis therapy was initiated after the diagnosis of pulmonary TB. One week after antituberculosis therapy, antiretroviral therapy was started. However, multiple mediastinal lymphadenopathies and chylothorax developed. Adequate drainage of the chylothorax, suspension of antiretroviral therapy, and continued antituberculosis therapy resulted in successful treatment and good outcome.

**Conclusions:**

Chylothorax is a rare manifestation of TB-associated IRIS in HIV-infected patients. Careful monitoring for development of IRIS during treatment of HIV-TB coinfection is essential to minimize the associated morbidity and mortality.

## Background

Patients with human immunodeficiency virus (HIV) infection are at risk for coinfection with *Mycobacterium tuberculosis *(TB) [1]. Highly active antiretroviral therapy restores the immunity of HIV-infected patients, but immune reconstitution inflammatory syndrome (IRIS) may develop [2,3]. We report a case of HIV and TB coinfection who developed chylothorax as a manifestation of IRIS after the initiation of antituberculosis and antiretroviral therapy within a one-week interval.

## Case Presenatation

**Figure 1:**
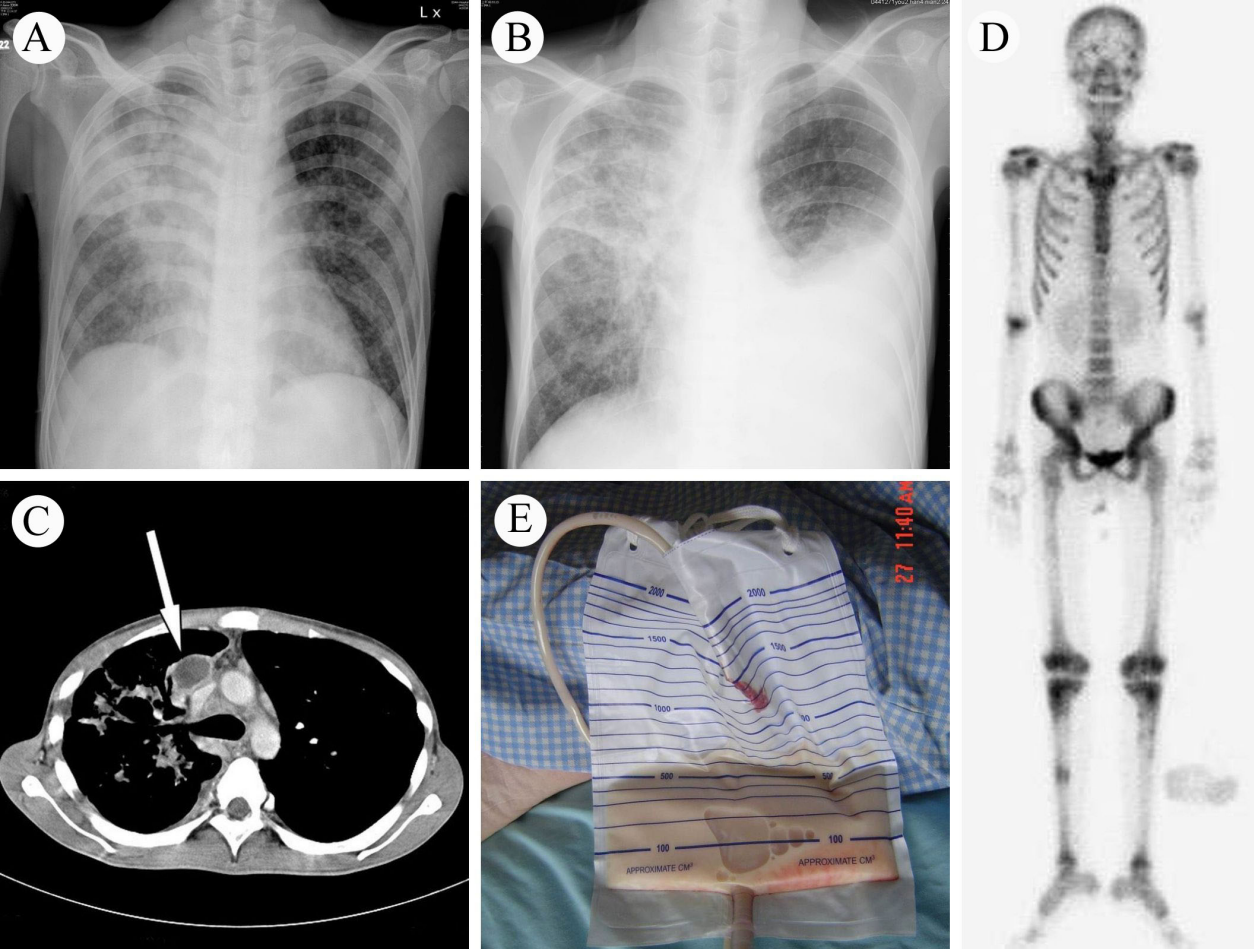
**The immune reconstitution inflammatory syndrome in a patient with human immunodeficiency virus and *Mycobacterium tuberculosis *coinfection**. (A) Chest X-ray on admission. (B) Chest X-ray 3 weeks after initiation of antiretroviral therapy. (C) Computed tomography of chest showed a large lymphadenopathy in mediastinum (arrow). (D) Tc-99 m methylene diphosphonate whole body bone scan showed multiple increased radioactive lesions in the axial and appendicular skeleton. (E) The drained chylous left pleural effusion.

A 25-year-old HIV-1-infected man presented with fever, productive cough, and body weight loss for 2 months. On admission, physical examination disclosed oral thrush and inspiratory crackles over his lung fields bilaterally. He had a CD4 cell count of 11 cells/μl and an HIV-1 viral load of 315,939 copies/ml. Chest X-ray revealed a consolidation in his right lung and multiple nodular infiltrations in the left lung field (Figure 1A). A sputum smear was positive for acid-fast bacilli and the sputum culture subsequently grew TB. He was treated with rifampin, isoniazid, ethambutol, and pyrazinamide initially, and his fever subsided gradually. Due to a strikingly low CD4 cell count, antiretroviral therapy with lamivudine, didanosine, and efavirenz was begun 1 week after starting antituberculosis chemotherapy. The patient became febrile again 4 days after the start of antiretroviral therapy. IRIS was suspected. His fever persisted despite prescription of oral prednisolone (1 mg/kg daily). The patient then suffered from progressive dyspnea, and a chest X-ray taken 3 weeks after starting the antiretroviral therapy demonstrated a massive, left-sided, pleural effusion (Figure 1B). Antiretroviral therapy was withheld in view of this potentially life-threatening manifestation of IRIS. Computed tomography of the chest disclosed several mediastinal lymphadenopathies (Figure 1C) and osteolytic lesions in the sternum, vertebra, and ribs. A Tc-99m methylene diphosphonate whole body bone scan showed multiple, increased radioactive lesions in his axial and appendicular skeleton (Figure 1D). Numerous acid-fast bacilli were found in a vertebral bone biopsy of T11. A pigtail catheter was inserted into his left pleural cavity, draining out a chylous effusion (Figure 1E). Pleural fluid analysis showed: total protein, 5.6 g/dl; lactate dehydrogenase, 154 IU/L; glucose, 100 mg/dl; total cholesterol, 66 mg/dl; and triglycerides, 1,230 mg/dl. Cytology of the pleural effusion was negative for malignant cells and cultures for bacteria, mycobacteria, and fungi were all negative. With continued antituberculosis therapy, the amount of chylous pleural effusion decreased. The drainage tube was removed 3 weeks later, after draining a total of 9,630 ml of chylous effusion. Corticosteroid was administered for 2 months. Antiretroviral therapy was re-started 1 month after removal of the drainage tube, and no further symptoms of IRIS were observed. The patient completed 12 months of antituberculosis therapy (2 months of rifampin, isoniazid, ethambutol, and pyrazinamide; 10 months of rifampin and isoniazid). There were no symptoms of relapse at follow-up visits over the next 6 months.

## Conclusions

IRIS is a paradoxical deterioration of the clinical status after the initiation of antiretroviral therapy. It is generally self-limiting or responds to the administration of non-steroidal anti-inflammatory drugs or corticosteroids. In patients with HIV and TB coinfection, paradoxical TB-IRIS has been observed in 8-43% of HIV-infected patients starting antiretroviral therapy while on TB treatment [3]. The risk of TB-associated IRIS in HIV-infected patients is higher for those with low CD4 cell counts and high HIV viral loads, and when antiretroviral therapy is initiated early in the course of antituberculosis treatment [3]. The optimal timing for the initiation of antiretroviral therapy after starting TB treatment is not known. Some experts suggest that the timing of initiation of antiretroviral therapy should be based on CD4 cell counts and the initiation of antiretroviral therapy should be delayed for 2 to 8 weeks in patients with a CD4 cell count of <200 cells/μl [4]. However, increased risk of developing further acquired immune deficiency syndrome-defining events or death in patients with a CD4 cell count of <100 cells/μl justify recommendations to start antiretroviral therapy as soon as possible in severely immunosuppressed HIV-infected patients coinfected with TB [5].

Chylothorax and chylous ascites are caused by the leakage of chyle into the pleural space and peritoneum due to rupture or obstruction of thoracic duct, respectively. Triglyceride levels >110 mg/dl, low cholesterol level, and the presence of chylomicrons in pleural effusion or ascites are diagnostic of a chylothorax or chylous ascites [6]. Chylothorax may occur as a complication of TB in patients with HIV infections [7]. In patients with initial recovery after antituberculosis treatment and then a subsequent deterioration after starting antiretroviral therapy, the presence of a resistant strain of TB should be excluded before establishing a diagnosis of IRIS. Rare case reports of chylothorax or chylous ascites as a manifestation of *Mycobacterium avium complex*-associated or TB-associated IRIS in HIV-infected patients have been described [8-10]. In advanced HIV infection, chylothorax may also be caused by infiltration of the thoracic duct by Kaposi sarcoma [11]. Tissue biopsy or cultures are required for differential diagnosis.

In our patient, chylothorax developed possibly due to the enlargement of mediastinal lymph nodes, which obstructed the thoracic duct flow and resulted in chyle leakage into the pleural space. While drainage relieved respiratory distress, failure to prevent the continual loss of chyle can lead to life-threatening consequences including hypovolemia, malnutrition, and electrolyte imbalance. The administration of corticosteroid did not halt the progressive increase in chylous pleural effusion in our patient. With suspension of antiretroviral therapy, continued antituberculosis therapy, corticosteroids, and adequate drainage, our patient was treated successfully without further sequela. This case illustrates the highly variable manifestations of TB-associated IRIS and suggests that close monitoring of clinical manifestations, especially in the first few weeks or months of antiretroviral therapy initiation, is essential to minimize the morbidity and mortality of TB-associated IRIS in HIV-infected patients.

## Abbreviations

HIV: human immunodeficiency virus; TB: *Mycobacterium tuberculosis*; IRIS: immune reconstitution inflammatory syndrome.

## Competing interests

The authors declare that they have no competing interests.

## Authors' contributions

JNL and CHL drafted the manuscript. YHC provided information and participated in writing the manuscript. LLC and SSL reviewed and revised the manuscript. HHL cared for the patient and conducted the study.

All authors have read and approved the final manuscript.

## Pre-publication history

The pre-publication history for this paper can be accessed here:

http://www.biomedcentral.com/1471-2334/10/321/prepub
